# Characterisation of Asian Snakehead Murrel *Channa striata* (Channidae) in Malaysia: An Insight into Molecular Data and Morphological Approach

**DOI:** 10.1155/2013/917506

**Published:** 2013-12-12

**Authors:** Li Min Song, Kaviarasu Munian, Zulkafli Abd Rashid, Subha Bhassu

**Affiliations:** ^1^Genomics and Evolutionary Biology Laboratory, Department of Genetics & Molecular Biology, Institute of Biological Sciences, Faculty of Science, University of Malaya, 50603 Kuala Lumpur, Malaysia; ^2^Zoology Branch, Forest Biodiversity Division, Forest Research Institute Malaysia (FRIM), 52109 Kepong, Selangor, Malaysia; ^3^Department of Fisheries Malaysia, Freshwater Fisheries Research Division, FRI Glami Lemi, Jelebu, 71650 Titi, Negeri Sembilan, Malaysia; ^4^Centre for Biotechnology in Agriculture Research, Division of Genetics & Molecular Biology, Institute of Biological Sciences, Faculty of Science, University of Malaya, 50603 Kuala Lumpur, Malaysia

## Abstract

Conservation is imperative for the Asian snakeheads *Channa striata*, as the species has been overfished due to its high market demand. Using maternal markers (mitochondrial cytochrome *c* oxidase subunit 1 gene (COI)), we discovered that evolutionary forces that drove population divergence did not show any match between the genetic and morphological divergence pattern. However, there is evidence of incomplete divergence patterns between the Borneo population and the populations from Peninsular Malaysia. This supports the claim of historical coalescence of *C. striata* during Pleistocene glaciations. Ecological heterogeneity caused high phenotypic variance and was not correlated with genetic variance among the populations. Spatial conservation assessments are required to manage different stock units. Results on DNA barcoding show no evidence of cryptic species in *C. striata* in Malaysia. The newly obtained sequences add to the database of freshwater fish DNA barcodes and in future will provide information relevant to identification of species.

## 1. Introduction

The freshwater snakehead *Channa striata* (Bloch, 1793), from the family Channidae, has a wide range of habitats ranging from rivers, swamps, ponds, canals, lakes, and land of rice fields. Their natural populations are extensively distributed across southern Asia, southern China, Indochina, and Sunda Islands [1–3]. This carnivore snakehead murrel, locally known as “Haruan,” is able to tolerate to adverse environments due to its hardiness and air-breathing capabilities assisted with a suprabranchial chamber, an air-breathing organ [4] which is unique to Channidae but exclusive in other freshwater fish families [5–7]. The local market demand on *C. striata* is greatly expanding due to its commercial value, agreeable flavor local food [3], and its postoperative medicinal application to enhance wound healing and reduce postoperative pain and discomfort [8]. A population structure of *C. striata* is urgently needed in handling this species in aquaculture and to provide spatial conservation value on this species.

To date, the exact taxonomic status at the level of species of the genus of *Channa* remains unclear as *C. striata* was placed as one of the “species complexes” under this mentioned genus [9]. This had raised the interest to investigate the phylogenetic relationship of the snakehead particularly the origin of the Asian snakehead and its evolution towards formation of different species of genus *Channa*. Previous studies showed that the occurrence of the Asian snakehead in southern Asia particularly in Southeast Asia was mainly due to phylogeographical influences [10]. The population structure of *C. striata* surveyed in Perak, Malaysia, was adduced to a historical Pleistocene event [11], strongly suggesting the coalescence of freshwater taxa. The Pleistocene event may explain the most historical coalescence of a species whereby multiple episodes of glaciations and deglaciations occurred with the accompanied of lowering and rising in sea water level happened approximately 2 million years ago. Historical Pleistocene glaciations with the maximum lowering sea water level greatly affected the land mass configuration and thus the biodiversity where Peninsular Malaysia, island of Borneo, Southern Thailand, Southern Indo china, Sumatera, and Java were interconnected [12]. The land bridge through the formation of Sunda River permitted the migration and gene flow of fauna between Peninsular Malaysia and the island of Borneo as the sea-water level rose that allowed submersion of this river system during the last Pleistocene Epoch [1, 13]. This phenomenon is shown by the genetic divergence pattern of *Hampala macrolepidota* [14], *Tor tambroides* [15], and *Barbonymus schwanenfeldii* [16] with the witness of nonsignificant genetic differentiation and sharing of the same haplotype between Peninsular Malaysia and the island of Borneo.

Environmental factors were recognized as an additive determinant on the phenotype of an organism. Human activities such as construction of dwellings that often involve forest destruction leading to sedimentation in the river coupled with biotic environmental factors such as food competition as a whole will influence the trait development for survival fitness. The physical changes as a response to polluted aquatic habitats were shown in previous studies in relation to deformity of fish morphology [17], deformation [18, 19], and fin erosion [20, 21]. The study of Roberts and Khaironizam [22] revealed that the mouth polymorphism of *Neolissochilus soroides* due to feeding behavior had occurred at sympatric population. This in turn led us to further investigate the pattern of phenotypic variation among allopatric populations of *C. striata* across Malaysia.

In this study, we adopted a mitochondrial cytochrome *c* oxidase subunit 1 (COI) gene as a genetic marker to assess the genotypic variation. This gene's comparatively slow mutation rate in relation to microsatellite markers and its maternal inheritance makes it possible to reveal any historical population dispersal pattern of a species. A population from Sarawak was included, as a representative of the island of Borneo to test the hypothesis of Pleistocene event on genotypic divergence pattern of *C. striata*. The morphological divergence pattern will be assessed through Truss Network Measurement [23]. Understanding the correlations between the genotypic and phenotypic divergence pattern would be informative to conservationists in proposing conservation assessments. The aim of this study is to characterize the pattern of population divergence of *C. striata* in order to provide a spatial conservation planning in different localities and investigate the possible hidden cryptic taxa of *C. striata* using a molecular species identification approach-DNA bar coding that was introduced by Hebert et al. [24]. This method for taxonomic identification avoids potential confusion surrounding accurate classification of discriminating morphological characters by focusing directly on the molecular level [25] and has been used as a universal method that is applicable across all eukaryote taxonomic groups [23, 26–32]. In relation to this study, mitochondrial (mtDNA) gene cytochrome *c* oxidase (COI) is the marker of choice [24]. Focusing on this standardized short sequence of DNA aims to benefit the taxonomist by assisting in the classification of known species as well as by identifying cryptic diversity, potentially leading to the discovery of new undescribed taxa [26, 33]. This approach appears to have been successful in differentiating among species within a range of animal groups [28, 34, 35] including freshwater and marine fishes [36, 37]. In this study, where the level of interspecies divergence will be taken into account, additional freshwater fishes of 26 fish species were chosen and where possible multiple individuals of each species were collected to examine diversity in the COI region at the interspecific and intraspecific level. Our suspected cryptic taxa of *C. striata* was sampled extensively across its geographical range in Malaysia in order to represent the extent of phylogeographical divergence that is likely to be present for this taxon across the region. This widely distributed taxon is thought to comprise a species complex [9] and has recently been found to occur in genetically divergent forms in SE Asia [10] and therefore is of particular taxonomic interest as it may show higher than average intraspecific divergence.

## 2. Materials and Methods

**Figure 1:**
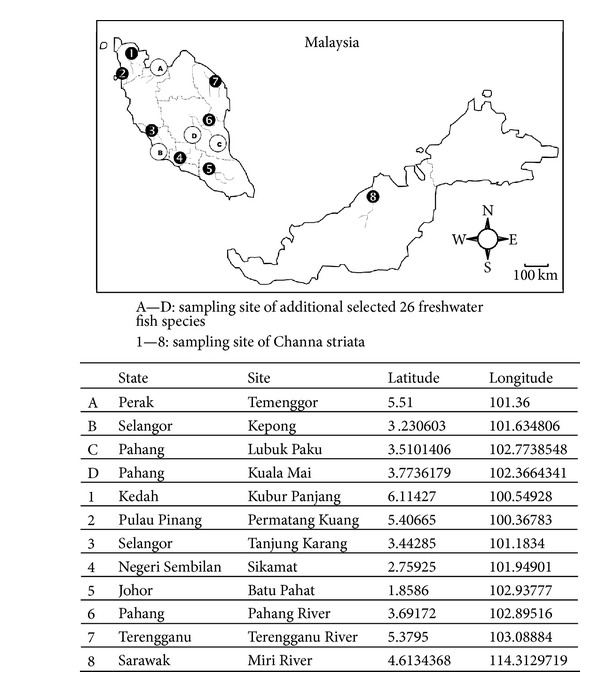
Sampling locations of 220 specimens of *Channa striata* and additional 83 freshwater fishes constituting selected 26 freshwater fish species used in this study. Exact latitude and longitude for each sampling sites are being recorded. Numbers 1–8 and alphabets A–D correspond to locations listed in Table 1.

A total of 220 specimens of *C. striata* representing eight locations of Kedah, Pulau Pinang, Selangor, Negeri Sembilan, Johor, Pahang, Terengganu, and Sarawak were collected and used in this study. Samples from Peninsular Malaysia were caught by simple fishing methods such as nets and samples from Sarawak were purchased from local market. These fish samples were collected by Freshwater Fisheries Research Division of Jelebu (Department of Fisheries Malaysia, Freshwater Fisheries Research Division, FRI Glami Lemi, Jelebu, Titi, Negeri Sembilan, Malaysia). Collection was made from 8 November 2006 to 27 August 2007. The samples from Sarawak were purchased by the author on 26 March 2011. In the case of DNA bar coding approach, an additional 83 fishes were sampled at Pahang (Kuala Mai and Lubuk Paku), Selangor (Kepong), and Perak (Temenggor), representing members of 26 previously described species from 23 genera, 7 families, and 3 orders (see Figure 1 for sampling location and details). Multiple individuals per species were collected where possible to provide some indication of the level of intraspecific variation present in the DNA bar coding fragment. In particular, 43 *C. striata* individuals with four to ten individuals as representatives from each eight sites across a wide geographical range in Malaysia were considered in DNA bar coding to provide an estimate of the likely extent of intraspecific divergence present in this species and to investigate possible cryptic taxa that might occur across Malaysia (see Table 1 for selected fishes used in DNA bar coding). These 43 *C. striata* samples were also genetically surveyed for population study in the case of examining amount of gene flow and genetic differentiation. All additional 83 fishes were caught by electrofishing, and all the collected samples in this study were positively identified to species level at the time of collection. The GPS location of collection sites was recorded. Muscle tissues of each sample collected were stored individually in absolute ethanol prior to genetic analysis, and voucher specimens were preserved in absolute ethanol and lodged at the Repository Store located at Freshwater Fisheries Research Division of Jelebu. Ten vouchers samples of *C. striata* from Sarawak were kept frozen in a −80°C freezer located at laboratory of University of Malaya (see Figure 1 for *C. striata* samples used in both molecular and morphometric of population study).

**Figure 2:**
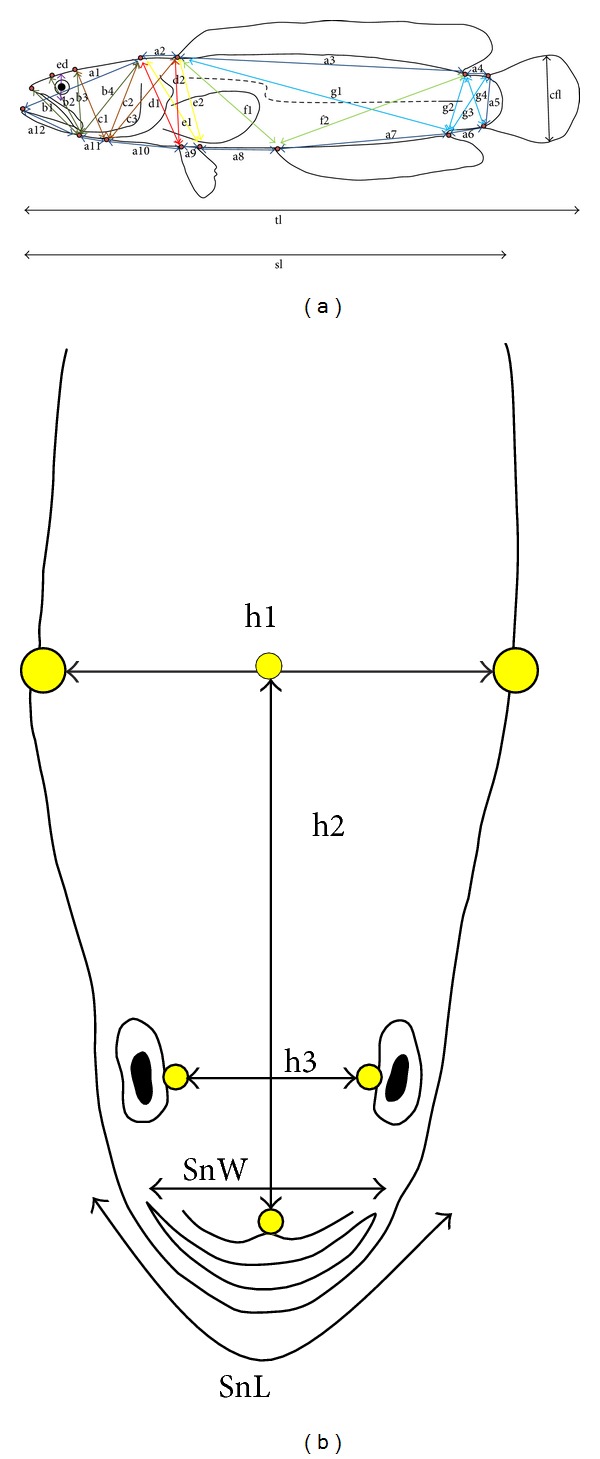
Schematic diagram of 38 morphometric measurements of *Channa striata* (total length (tl), standard length (sl), caudal fin length (cfl), eyes diameter (ed), snout width (SnW), and snout length (SnL) and measurement of each body part (a1–a12, b1–b4, c1–c3, d1-d2, e1-e2, f1-f2, g1–g4, and h1–h3)) based on the Truss Network Measurement [23].

For samples brought back to the laboratory, 38 morphometric measurements were taken to the nearest centimeter based on Truss Network Measurement (see Figures 2(a) and 2(b) for all the linear measurements) [22]. A box-plot analysis was done using SPSS software version 16.0 (SPSS, Inc., Chicago IL) to exclude the extreme measurements which indicate outlier. Prior to multivariate discriminant analysis, transformation of size-independent shape variables was carried out using SPSS software version 16.0 (SPSS, Inc., Chicago, IL, USA) with the formula of *M*_adj_ = *M*(*L*_*s*_/*L*_*o*_)^*b*^ [38], where *M*_adj_ is the transformed morphometric measurement to be used in subsequent multivariate analysis, *M* is the original morphometric measurement, *L*_*s*_ is the overall mean of standard length obtained from all samples for each variable, *L*_*o*_ is the standard length of the fish, and *b* is the slope of regression of log⁡*M* over log *L*_*o*_. Parameter *b* is calculated to check the effect of size on the measurement and transformed *M*_adj_ variables were used in the following multivariate discriminant analysis when the transformed variable did not show significant correlation with standard length.

Multivariate discriminant analysis was then carried out using software STATISTICA version 8 (StataCorp. 2003. *Stata Statistical Software: Release 8.* College Station, TX: StataCorp LP) based on transformed morphometric measurements. This analysis highlighted the two traits which had the greatest contribution in discriminating populations and the pattern of variation will be illustrated in two-dimensional graph. Specifically, there are unobserved patterns of variation lying behind each observed measure and multivariate discriminant analysis aims to group those underlying variation which are correlated to each other under the same root. The pattern of variation underlined by each root can be seen in two-dimensional graph in which those populations located at positive region of each root acquired unique extreme measurements of the two highly correlated traits that could discriminate them from other population located at negative region of the same root. These two discriminant traits will be referred to the “List of Standardized Coefficients of Canonical Variables” which will highlight the two highly correlated traits that had the greatest contribution of the variation for each root. These discriminant traits are those traits with the two top coefficient values in the mentioned list assigned to the “positive” and “negative” sign of the value. The comparative small or comparative large measurement of the trait in relation to others will then be referred to the “negative” and “positive” sign of the coefficient value, respectively [38].

Initially, a multivariate discriminant function which group the populations based on similarity on certain characters was performed with all the populations from Peninsular Malaysia. Secondly, in order to have a balance sample size for data analysis, populations with overlapping centroid mean are considered as single group and subsets of each group together with Sarawak population were included in further multivariate discriminant analysis.

A total of 43 *C. striata* specimens were used in this analysis. Between four to ten samples representing each site (sites 1–8; refer to Figure 1) were used in genetic analysis. Once morphometric measurement was taken, approximately 2 cm × 1 cm of tissue samples were dissected and preserved in absolute ethanol prior to genetic analysis and stored at −80°C freezer for long-term storage. Genomic DNA was extracted from each tissue sample using AxyPrep Genomic DNA Extraction Kit according to the animal tissues spin protocol provided by the company (AxyPrep Genomic DNA Extraction Kit website: http://www.axygenbio.com/collections/vendors?page=2&q=Axygen). Polymerase chain reactions (PCRs) were then performed to obtain ~700 bp COI amplicons with the primer pair FishF1: 5′TCAACCAACCACAAAGACATTGGCAC3′ and; FishR1: 5′TAGACTTCTGGGTGGCCAAAGAATCA3′ [39]. PCR was performed with no modifications to standard PCR reaction. Successful amplification was determined through 1% agarose gel electrophoresis, with the products visualized by staining with ethidium bromide. After agarose gel electrophoresis, PCR products were abscised from the gel and purification was performed using AxyPrep Gel DNA Extraction Kit according to the provided protocol (AxyPrep Gel DNA Extraction Kit). After purification, the products were sequenced in both directions using the Big Dye version 3.1 chemistry following the manufacturer's instructions (Applied Biosystem). After sequencing reactions the sequencing products were purified by ethanol precipitation and analyzed on an ABI 3730 capillary sequencer (Applied Biosystems).

Prior to data analysis, nucleotide sequences were trimmed to a length of 582 bps to remove any areas where base calling was uncertain and then all sequence reads were checked against chromatograph data using Chromas Software (Technelysium Pty., Ltd.) and Mega Software version 4.1 [40]. The final alignment began at position 6552 of the complete mitochondrial genome of *Oncorhynchus mykiss* (GenBank accession number: NC_001717.1). All the sequences were submitted to the GenBank database with accession numbers HM156340–HM156344, HM156350–HM156372, HM156377–HM156390, HM156392, JF781170–JF781242, and JN695688 to JN695697, and to the Barcode of Life Database (BOLD: http://www.barcodinglife.com/ or [41])

To ensure that the desired COI region had been obtained, DNA sequences were aligned using the Basic Local Alignment Search Tool (BLAST) [41] to check for homology to other vertebrate COI sequences. In some cases, nontarget fragments such as nuclear mtDNA pseudo genes (numts) may be amplified during bar coding [42, 43]. To ensure that this was not a problem for the current data set, all sequences were screened for the presence of stop codons and insertion-deletion mutations using the software Mega version 4.1 [42]. The software DnaSP v5 [44] was used to summarize haplotype information. For DNA bar coding analysis, within the group, mean genetic distances were calculated within species, within genera and, where sampling was appropriate, within one family (Cyprinid) using the Kimura 2 Parameter (K2P) distance model [45] in the software Mega version 4.1. Interspecific K2P differentiation was also calculated for all possible species through pairwise comparisons using Mega software. Phylogenetic trees were constructed to illustrate the patterns of divergence among species and within species where multiple haplotypes, had been detected. Where multiple individuals possessed identical haplotypes, only one representative was included in the phylogenetic analyses. Two species, *Amia calva* (genBank accession number: EU524435.1) and *Atractosteus tristoechus* (genBank accession number: FN545592.1), were included as out-group taxa. A neighbour-joining (NJ) tree (Saitou and Nei, 1987) was constructed in Mega version 4.1 using the K2P distance model with 1,000 bootstrap replicates. Secondly, a Bayesian tree was estimated using MrBayes software [46]. For this analysis, parameters, not part of the default settings were a *4by4* nuclear model, an *nst* of 6, and an invariable site with gamma model, with analyses run for 200,000 generations of simulations under a Markov Chain Monte Carlo (MCMC) model. The first 25% of samples were discarded as burn-in and trees were sampled every 100 generations. For the population study at a genetic level, a haplotype network connected by all the haplotypes by mutational step was performed using software TCS version 1.21 [47]. The *F*_st_ value which determines the population differentiation was then calculated using software Arlequin version 3.11 [48]. Population comparison was performed by computing pairwise differences with number of permutation of 100 and significant level at *P* < 0.05.

## 3. Results 

A total of 126 samples obtained from 27 freshwater fish species were characterized for a 582-base-pair length of mtDNA COI sequence. All fragments aligned well with other COI barcodes in the GenBank database and showed no evidence of the presence of indels or in-frame stop codons that may have indicated that the regions were numts. Therefore, it was assumed that the nucleotide sequences generated here truly represented the mtDNA COI barcode region and were suitable for subsequent analysis.

**Figure 3:**
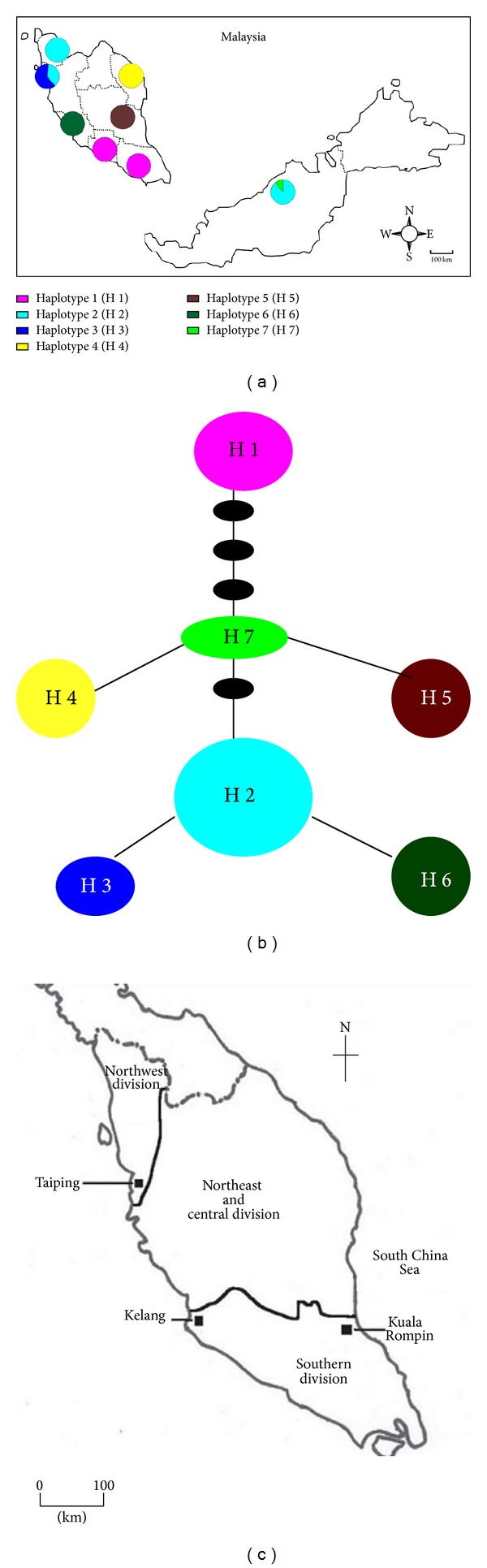
(a) Haplotype distribution of seven haplotypes generated from eight populations of *Channa striata* across Malaysia. (b) Haplotype network connecting all generated haplotypes; the size of each haplotype is proportional to the number of each individual of the corresponding haplotype; each line represents one mutational step. (c) Three main divisions of Peninsular Malaysia. Adapted from Mohsin and Ambak [1, 49] cited in [16].

For all 43 *C. striata* sequences, the aligned COI sequences consist of nine nucleotide variable sites contributing 1.55% of intra-species nucleotide variation. All the variable sites are parsimony informative. Overall, there are seven unique haplotypes (hap) (Figures 3(a) and 3(b)) identified from 43 *C. striata* specimens used in this study with high haplotype diversity value (*h* = 0.8018) being recorded. The haplotype distribution in Peninsular Malaysia is parallel to the partition division in Mohsin and Ambak (Figure 3(c)) [1, 49] with no overlapping of haplotype between the three regions of the north, central, and south division of Peninsular Malaysia. However, there are three unique haplotypes (hap4, hap5, and hap6) identified with no repetition in the three locations within the central division of Peninsular Malaysia. Hap2 is the most common haplotype and interestingly this haplotype sequence overlapped West (Kedah and Pulau Pinang) and East Malaysia (Sarawak) sequences. The population structure of *C. striata* was obtained by using the *F*_st_ value (Table 2) from the underlying population differentiation with the benchmark of *P* < 0.05 as the borderline of significant difference. In this study, there are no significant differences (*P* < 0.05) between populations which are in the north coast (Kedah and Pulau Pinang) and population in the south coast (Negeri Sembilan and Johor). However, the degree of population differences is not correlated to the geographical distance due to the absence of significant difference between East Malaysia (Sarawak) and the north coast of West Malaysia (Kedah and Pulau Pinang) which are separated by South China Sea.

**Figure 4:**
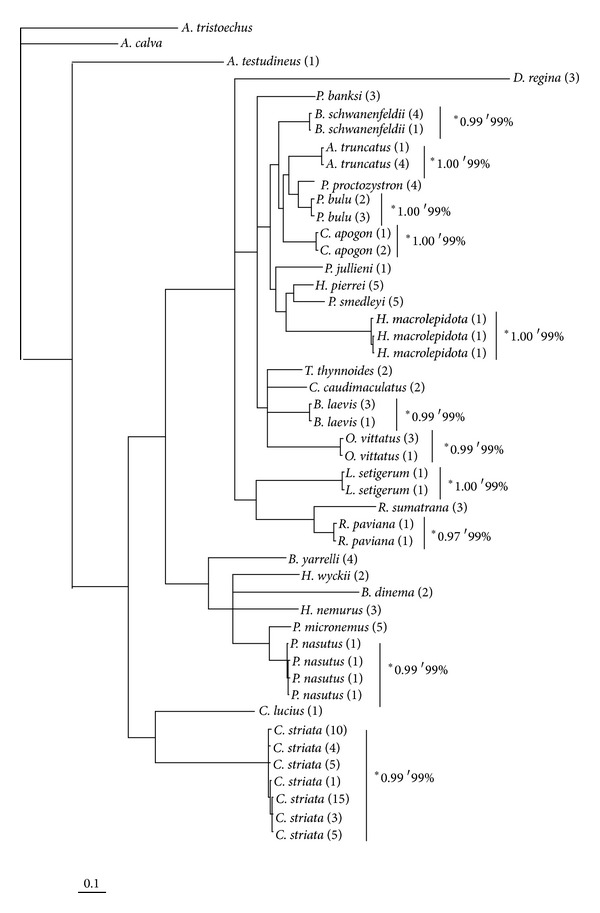
Phylogenetic tree of mtDNA COI barcodes of 27 fish species with the number of individuals of each haplotype listed in brackets after the species name. Posterior probability and bootstrap among different haplotypes of same species are shown by * and ′, respectively.

For the DNA bar coding results among the 27 fish species surveyed, 46 unique haplotypes were detected with no haplotypes shared among different species. The K2P divergences are presented in Table 3 and summarized within and among taxonomic levels in Table 4. The divergence between individuals within each species was low with K2P distances ranging from 0.00% to 0.90%, whereas average congeneric distance ranged from 1.70% to 10.10%. Despite the relatively intensive intraspecific sampling of *C. striata*, this species did not show the highest level of intraspecific divergence, which was instead observed among the two *Rasbora paviana* collected from sites A and B (0.90%). The maximum divergence among *C. striata* individuals was 0.60%, less than the mean divergence between *C. striata* and the congeneric *C. lucius* (1.70%), indicating that the divergence between these species was comparable to the divergence observed at the same taxonomic level among other congeneric groups. On average, there was almost 38-fold more variation among congeneric species (mean divergence of 6.03%) than among con-specific individuals (mean divergence of 0.16%), with the minimum congeneric divergence (1.70%) more than twice the maximum intraspecific divergence (0.90%). This pattern of divergence is reflected in the phylogenetic tree (Figure 4), where all haplotypes from the same species are clustered together with a high statistical support under both NJ and Bayesian inference methods. The mean genetic distance among the 18 species sampled within the Cyprinid family was 15.7%, almost 3-fold greater than the mean divergence observed among genera.

**Figure 5:**
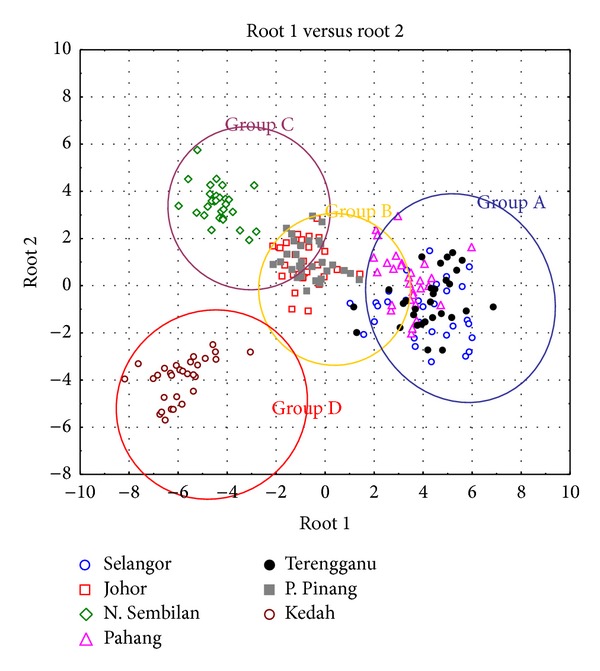
Multivariate discriminant analysis based on 7 locations plotted on root 1 and root 2 axes. Grouping of Groups A–D was based on the overlapping pattern of each population centroid mean. The two discriminant traits that contribute most to the variance underlined by each root are referred to as the coefficient values listed in Table 6.

**Figure 6:**
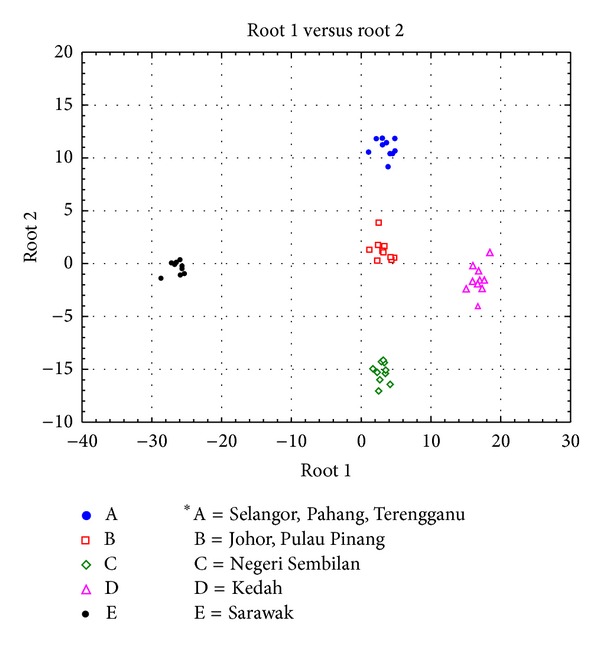
Multivariate discriminant analysis based on 8 locations plotted on root 1 and root 2 axes. The two discriminant traits that contribute most to the variance underlined by each root are referred to as the coefficient values listed in Table 6.

Discriminant analysis based on 30 successfully transformed morphometric measurements had successfully discriminate locations within Peninsular Malaysia as well as between the West and the East of Malaysia. For the discriminant analysis within Peninsular Malaysia, four distinct groups had been discovered with nonoverlapping centroid mean with each other as shown in the two-dimensional graph of discriminant function (Figure 5). There are group of multiple populations consists of Selangor, Pahang, and Terengganu, group of two populations of Pulau Pinang and Johor, group of single population of Negeri Sembilan, and another group of single population of Kedah. These resulted groups are further assigned as groups A, B, C, and D, respectively, for further discriminant analysis which include Sarawak as group E as a representative population from East Malaysia. The eigenvalues are the greatest on root 1 and root 2 of the discriminant function among populations in Peninsular Malaysia and hence contributing 58.54% and 18.49% of the total variance, respectively, in discrimination of morphometric character. Refer to Table 5 for details. The greatest contribution of character to the root 1 was made by measurement of c2 and e1 which are located at posterior part of the head, whereas the greatest contribution to root 2 was contributed by measurement of c3 and d2 which are also located at posterior part of the head. Specifically, the group of Selangor, Pahang, and Terengganu was characterized by comparatively small c2 measurement and comparatively large e1 measurement in comparison to other locations; the group of Pulau Pinang, Johor, and Negeri Sembilan was characterized by comparatively small c3 measurement and comparatively small d2 measurement in comparison to other locations. This data suggested that *C. striata* population in Peninsular Malaysia was significantly discriminated from each other by distinct size of the head. When compared to Sarawak population using discriminant analysis, the discriminant function graph shown in Figure 6, root 1 had the greatest variance (74.42%) to populations of Peninsular Malaysia and the Sarawak population (group E) formed a distinct group and is well discriminated by root 1. The greatest contribution of measurement to root 1 was made by b1 measurement and e2 measurement and hence West Malaysia populations were uniquely characterized by a comparatively large b1 measurement and comparatively large e2 measurement which are the measurements of the size of the mouth and posterior part of the head, respectively, in contrast to population from East Malaysia (refer to Table 6 for details).

Briefly, the population molecular divergence pattern discovered in this research study did not show a match to morphological divergence pattern. Populations from East Malaysia (Sarawak) and West Malaysia (Kedah and Pulau Pinang) are genetically alike with the evidence of sharing of the same haplotype (hap2) and nonsignificant *F*_st_ value, whereas, morphologically, the island of Borneo in this study case was discriminated from Peninsular Malaysia as witnessed by the second stage of multivariate discriminant analysis.

## 4. Discussions

In our study, in order to make any judgment of unidentified cryptic taxa in *C. striata*, validation of COI barcode in discriminating freshwater fish species in Malaysia is required. Among all 27 freshwater fish species surveyed for DNA bar coding, divergence among these 27 species surveyed here was relatively high, suggesting that the COI marker was able to differentiate between the Malaysian freshwater fish species examined in this study. In every case where multiple haplotypes were sampled from a single species, all haplotypes were closely related, with no haplotypes common among more than one species and strong statistical support that each individual species sampled was monophyletic. Previously bar coding studies have reported the same incidences of shared haplotypes among species (see [37]). These cases are probably most often associated with sister taxa where the time since speciation has not been sufficient for lineage sorting and divergence to result in monophyly, although introgressive hybridization among more divergent taxa has also been observed [26, 37]. The finding here that there were no haplotypes common to multiple species is encouraging from a bar coding perspective; however, greater taxonomic sampling of more closely related taxa may yet uncover shared haplotypes and introgressive hybridization among Malaysia's many freshwater fish species. Ideally, the appropriate threshold level for determining interspecific versus intraspecific should be high enough to separate samples that are likely to belong to different species, but not that representing different haplotypes of the same species. Hebert et al. [26] proposed that the standard threshold level for designating two taxa as different species should be 10X the mean intraspecific variation for the group under study. In the current study very low mean divergence was observed among individual haplotypes belonging to the same species (mean intraspecific divergence of 0.16%, maximum divergence 0.90%), and in contrast mean overall congeneric divergence was 6.03%. Overall, the minimum congeneric distance (1.70%) was almost 11-fold higher than the average variation among con-specific individuals (0.16%), thus clearly dividing the haplotypes into intraspecific and interspecific groups under Hebert et al.'s [26] 10X benchmark. This ratio of congeneric to average con-specific variation parallels the findings of other DNA barcode studies on animals (13X-fold higher in Neotropical bats [34], 18X-fold higher in birds [26], and 27X-fold higher in Canadian freshwater fish [37]. In the current study, taxonomic sampling was relatively sparse, and it is likely that as more Malaysian freshwater species are surveyed for each genus within families, the average level of congeneric genetic distance may decrease. Nonetheless, the data presented here implies that this value is likely to remain higher than the 10X threshold. It is also likely that, within species, higher mtDNA COI divergence may be present among individuals sampled across the entire species in its natural geographical range. In this case, the 10X method may be employed to investigate if the level of divergence present may represent the presence of cryptic speciation or was simply a result of regional phylogeographical structuring [27, 50]. This study employed extensive geographical sampling of one species of freshwater fish in Malaysia, *C. striata*, and for this taxon intraspecific divergence was 0.60% less than the interspecific divergence observed among *C. striata* and *Channa lucius* (1.70%). Although when considered independently from divergences among the entire dataset the pattern of divergence within the *Channa* does not conform to the 10X threshold for species delineation proposed by Hebert et al. [26], and this may be a result of sparse taxonomic sampling, as greater sampling effort that included more *Channa* species is likely to increase estimates of average congeneric divergence among this group. Furthermore, this study reveals that although highly divergent populations of *C. striata* have been found in other parts of its natural geographical distribution [10], there is no evidence of cryptic diversity in this species in Malaysia. While generating these 126 barcodes in revealing unidentified cryptic taxa, the successfully identified barcodes will then aid in future study to act as a reference, so that in the future when an unknown taxon is sampled it can be identified by matching its sequence against a DNA barcode database (i.e., BOLD). This is complementary to the aim of the DNA bar coding approach which is to assign a unique DNA barcode or set of DNA barcodes to a particular species, in such a way that the same barcode will never be common among two species [24]. Previous studies on the same specimens from similar locality in Peninsular Malaysia reveal the possibility of cryptic taxa of *C. striata* in Negeri Sembilan through the molecular dispersal patterns of *C. striata* using rapidly evolving microsatellite marker [51].

In the population study, gene flow is predicted to occur in adjacent populations such as between Kedah and Pulau Pinang and between Negeri Sembilan and Johor which recorded a nonsignificant *F*_st_ value (*P* < 0.05). This could be explained by the possibility of migration of fish across adjacent drainage systems due to flooding [52]. This then follows the one-dimensional stepping stone models [52] that allow migration to adjacent population. This is typical of the migration pattern of *Acrossocheilus paradoxus* found in the model in Taiwan [52].

Physical separation of an inhabited geographical range allows a particular organism to acquire different genetic structures. It appears that freshwater species is a casualty of physical barriers such as mountains and dam construction effectively disconnecting them from the drainage basin. An interesting finding in this study was the sharing of hap2 between West Malaysia (Kedah and Pulau Pinang) and East Malaysia (Sarawak) and the nonsignificant population pairwise difference among these two isolated locations, indicating the presence of gene flow and migration of fish among the mainland of Peninsular Malaysia and the island of Borneo during the historical event of Pleistocene Epoch. This epoch encountered with the multiple scenarios of glaciations and deglaciations with the resulted rising and lowering sea-water level and hence periodically changes the exposed land mass. The historical event during Pleistocene with the accompanied fluctuation in sea level could explain the incomplete divergence of *C. striata* between West and East of Malaysia. Although physical barriers could eventually separate the allopatric populations in central region in Peninsular Malaysia, the historical event of Pleistocene as a whole will explain the historical genetic divergence pattern of *C. striata* in Malaysia. Our finding with overlapping haplotype distribution between the West and the East Malaysia indicates that the fluctuating sea-water level during Pleistocene played a predominant role in permitting genetic exchange between the mainland of Peninsular Malaysia and the island of Borneo through the formation of Sunda River which allow migration of *C. striata* and thus permitting gene flow.

The Pleistocene glaciations [12] period happened approximately 250,000 years ago. The mainland of Peninsular Malaysia and the island of Borneo together with southern Thailand, southern Indo-China and Sumatra was once part of the submerged Sunda Shelf [1]. Because of the effect of maximum sea-water lowering during Pleistocene, the mainland of Peninsular Malaysia and the island of Borneo was once connected through the formation of Sunda River which is rich in fish diversity due to water draining out from the eastern coast of Peninsular Malaysia and western coast of Borneo and Sumatra [1, 13]. This historical land bridge allowed the migration of fish species between these regions, as in this case *C. striata*, until the end of the Pleistocene era when this great river system was submerged due to rising in sea-water level as a result of the deglaciations period [1, 13]. Recent increase in sea-water level followed by the formation of South China Sea is suspected to have isolated the island of Borneo from the mainland of Peninsular Malaysia. It is possible that, once the hap7 had colonized, the east and the south of Peninsular Malaysia and could have mutated spatially. The overlapping haplotype pattern between Negeri Sembilan and Johor suggested that gene flow was most likely to happen between them laterally, which further suggested the lateral dispersal behavior of *C. striata*. It is suspected that hap1, hap4, and hap5 were formed after separation of Peninsular Malaysia from the island of Borneo due to these haplotypes not being any haplotype leading towards formation of those Sarawak's haplotypes (hap2 and hap7). Hence, we predict that complete population divergence will occur between West and East Malaysia after a long period of separation by South China Sea. Indeed, in our study, it is believed that there is no colonization of *C. striata* on the island of Borneo and it is expected that the population in Sarawak is the migrated population from the mainland of Peninsular Malaysia. This is supported by previous study of Adamson et al. [10] which hypothesized that the evolution of majority of the *Channa* species including *C. striata* happened in South East Asia, specifically, the north part of Indochina. The biogeographically structure between India and South East Asia coupled with historical climatic changes caused fish flow through southeastern route from India. Geographically, the population on the east coast of Peninsular Malaysia which is closer to the island of Borneo has a greater tendency to migrate than the west coast due to the draining of water from the east of mainland towards Sunda River. However, the population group of Kedah, in the northwest of Peninsular Malaysia, may have migrated closer to the northern region of Peninsular Malaysia and then eventually flowed through the river streams draining out towards Sunda River during Pleistocene glaciations. This hypothesis is supported by previous study of Esa et al. [15] on *Tor tambroides* which shows a possibility of a migration route between northern Peninsular Malaysia and Borneo due to the same haplotype between populations from Batang Ai, Sarawak, and Perak, northern Peninsular Malaysia. In short, historical Pleistocene glaciations and deglaciations accompanied with changing in land mass configuration will describe the historical coalescence of a freshwater species such as in previous studies on *Hampala macrolepidota* [17], *Tor tambroides* [15], and *Barbonymus schwanenfeldii* [16].

The morphology divergence pattern of *C. striata* was not correlated to genotypic divergence pattern due to the morphology of certain terrestrial and marine animal having an additive effect of genetic components and environmental factors. A simple explanation would be that there is no connection between variation at the single gene investigated and variation in body size and shape.

## 5. Conclusion

In summary, the current dataset did not include sample's sex details due to the evidence of the absence of cytological distinguishable sex chromosome [53]. The morphological divergence pattern of *C. striata* is more appropriate to be significantly affected by environmental heterogeneity that influence the development of certain morphology characters and such development changes might be a response to adaptive survival in certain harsh environmental conditions. These developmental changes need not be correlated with the underlying genetic changes due to morphology, as it is the additive effect of the genetic component and environmental conditions that induce changes in certain observable character for survival fitness. Although *C. striata* is able to survive in harsh environments, conservation planning on this species is required due to its highly commercialize value which has led overfishing to further reduce its effective population size. In this study, we provided information on spatial pattern of conservation planning on this species. Although our study did not reveal any high diversity population in one of the surveyed locations, however, conservation of this species at its natural variation level is required as *C. striata* forms a diverse group of taxa across Malaysia with seven haplotypes distributed across Malaysia and the majority of populations show significant differences among each other. Our study also revealed that there are two groups with populations within each group which are genetically alike and hence exchangeable for stock management purpose; there are group of Negeri Sembilan and Johor and another group of Kedah, Pulau Pinang, and Sarawak. The current data compiled did not reveal any commercial characters such as larger body size, which will produce more flesh, but the morphological divergence pattern of *C. striata* indicates that environmental factors had a significant impact on the evolution of certain traits. Our molecular divergence pattern is not congruent to the pattern of morphology variation due to environmental conditions playing a significant role in population morphology divergence or simply the genetic variation across the whole genome would have to be investigated if we wanted to explore the connections between genotype and phenotype. The divergence patterns of both genotypic and phenotypic data reveals that spatial conservation assessment on *C. striata* is needed to conserve this species. Spatial conservative management could be applied to maintain the genetic diversity and to improve aquaculture quality.

## Figures and Tables

**Table 1:** List of 27 selected freshwater fishes species used in this study for DNA barcoding. Numbers and letters under location refer to Figure 1.

Order	Family	Genus	Species	Number of individuals	Local name	Location
Cypriniformes	Cyprinidae	Amblyrhynchicthys	*Amblyrhynchicthys truncatus *	5	Tempuling	(C)
		Barbichthys	*Barbichthys laevis *	4	Bentulu	(C)
		Barbonymus	*Barbonymus schwanenfeldii *	5	Lampam Sungai	(C)
		Chirrinus	*Cirrhinus caudimaculatus *	2	Selimang batu	(D)
		Cyclocheilichthy	*Cyclocheilichthys apogon *	3	Temperas	(D)
		Devario	*Devario regina *	3	Danio	(A)
		Hampala	*Hampala macrolepidota *	3	Sebarau	(C, A)
		Hypsibarbus	*Hypsibarbus wetmorei *	5	Kerai Kunyit	(C)
		Luciosoma	*Luciosoma setigerum *	2	Nyenyuar	(D)
		Osteochilus	*Osteochilus vittatus *	4	Terbul	(C)
		Poropuntius	*Poropuntius smedleyi *	5	Tengas daun	(B)
		Probarbus	*Probarbus jullieni *	1	Temoleh	(C)
		Puntioplites	*Puntioplites bulu *	5	Tengalan	(D)
			*Puntioplites proctozystron *	4	Kerengkek	(C)
		Puntius	*Puntius banksi *	3	Tengas	(B)
		Rasbora	*Rasbora sumatrana *	3	Seluang	(C, D)
			*Rasbora paviana *	2	Seluang	(A, B)
		Thynnichthys	*Thynnichthys thynnoides *	2	Lomah	(D)

Perciformes	Anabantidae	Anabas	*Anabas testudineus *	1	Puyu	(D)
	Channidae	Channa	*Channa striata *	43	Haruan	(1, 2, 3, 4, 5, 6, 7, 8)
			*Channa lucius *	1	Bujuk	(B)

Siluriformes	Bagridae	Hemibagrus	*Hemibagrus wyckii *	2	Baung kelulang	(D)
			*Hemibagrus nemurus *	3	Baung	(A)
	Pangasiidae	Pangasius	*Pangasius nasutus *	4	Patin buah	(D)
		Pseudolais	*Pseudolais micronemus *	5	Patin juara	(D)
	Siluridae	Belodontichthys	*Belodontichthys dinema *	2	Gerahak	(D)
	Sisoridae	Bagarius	*Bagarius yarrelli *	4	Kenderap	(D)

3 Order	7 families	23 genus	27 species	126 fishes	27 species	12 sites

**Table 2:** Pairwise population distance represented by *F*_st_ value with bold and “*” indicates significant *F*_st_ (*P* < 0.05).

Site	(1)	(2)	(3)	(4)	(5)	(6)	(7)	(8)
(1)								
(2)	0.00000							
(3)	1.00000*	1.00000*						
(4)	0.95455*	0.95455*	0.45205					
(5)	1.00000*	1.00000*	1.00000	0.89992*				
(6)	1.00000*	1.00000*	1.00000*	0.93478*	1.00000*			
(7)	1.00000*	1.00000*	1.00000*	0.81250*	1.00000*	1.00000*		
(8)	0.95271*	0.95271*	−0.12150	0.40239	0.92243*	0.92817*	0.77941*	

Number of site (1): Johor, (2): Negeri Sembilan, (3): Kedah, (4): Pulau Pinang, (5): Terengganu, (6): Pahang, (7): Selangor, and (8): Sarawak.

**Table 3:** Pairwise genetic K2P distances among taxa. Intraspecific values shown in bold and marked by asterix, where multiple distances were present between intraspecific haplotypes the largest value is shown.

	[1]	[2]	[3]	[4]	[5]	[6]	[7]	[8]	[9]	[10]	[11]	[12]	[13]	[14]	[15]	[16]	[17]	[18]	[19]	[20]	[21]	[22]	[23]	[24]	[25]	[26]	[27]
[1] *B. schwanenfeldii *	*0.10																										
[2] *A. truncatus *	11.30	*0.10																									
[3] *B. laevis *	14.80	14.50	*0.10																								
[4] *O. vittatus *	17.40	18.40	16.50	*0.10																							
[5] *H. wetmorei *	11.10	11.80	11.50	15.70	*0.00																						
[6] *P. proctozystron *	10.40	9.30	13.70	18.40	10.70	*0.00																					
[7] *H. macrolepidota *	18.50	18.80	17.80	20.30	15.10	16.50	*0.50																				
[8] *L. setigerum *	20.30	19.50	20.40	22.00	18.80	18.70	21.30	*0.20																			
[9] *B. yarrelli *	23.80	23.10	22.40	26.60	22.60	24.70	27.00	27.50	*0.00																		
[10] *R. sumatrana *	19.90	18.50	17.90	21.20	18.80	19.30	21.90	21.40	23.00	*0.00																	
[11] *P. micronemus *	23.50	24.00	23.80	25.90	24.40	23.80	24.70	24.10	18.00	23.50	*0.00																
[12] *P. jullieni *	14.30	14.80	13.60	14.40	11.90	12.50	16.30	21.60	22.10	21.30	24.50	*N/A															
[13] *P. nasutus *	24.10	22.30	23.40	25.00	24.00	23.50	24.00	23.90	18.00	21.80	8.00	25.30	*0.30														
[14] *T. thynnoides *	14.30	14.90	12.90	15.40	14.20	12.70	17.40	18.80	23.10	19.60	23.10	14.90	21.30	*0.00													
[15] *H. wyckii *	25.10	21.40	22.90	26.60	23.80	24.00	24.40	24.40	19.30	24.30	17.20	25.20	17.70	24.10	*0.00												
[16] *C. caudimaculatus *	13.60	15.90	12.50	16.30	15.10	13.70	20.70	20.20	23.80	17.20	22.30	16.80	22.50	12.40	22.80	*0.00											
[17] *C. apogon *	12.70	11.30	12.90	18.20	11.60	10.40	17.70	18.30	22.50	18.40	21.80	13.60	23.10	13.80	24.20	13.20	*0.50										
[18] *A. testudineus *	24.70	26.20	25.20	27.70	25.70	26.70	28.70	25.00	29.90	25.40	25.80	25.20	24.60	24.20	26.20	22.50	26.10	*N/A									
[19] *B. dinema *	23.60	25.60	24.60	25.80	23.50	25.10	25.60	25.30	21.00	23.30	22.20	26.60	21.40	25.80	24.10	22.60	24.70	26.30	*0.00								
[20] *P. bulu *	11.10	9.90	14.60	19.10	12.20	6.30	17.20	18.60	24.20	19.00	22.80	14.50	22.00	12.00	22.60	13.80	10.30	24.90	26.40	*0.50							
[21] *C. striata *	24.50	27.30	22.90	25.30	26.10	26.40	27.80	25.50	24.40	26.10	23.40	26.50	23.70	22.00	22.50	25.20	25.80	25.30	23.40	25.50	*0.60						
[22] *R. paviana *	19.70	18.50	17.90	21.60	18.20	19.00	20.00	18.70	24.20	14.30	22.40	19.80	20.90	18.10	24.00	16.50	17.60	26.20	24.60	18.00	25.40	*0.90					
[23] *H. nemurus *	23.60	23.60	22.20	24.70	22.30	24.00	26.00	25.40	20.20	25.10	15.40	22.60	17.90	23.80	16.80	23.50	22.40	30.40	24.10	24.40	24.00	25.40	*0.00				
[24] *D. regina *	27.50	24.50	26.40	29.00	26.40	25.90	28.50	31.20	30.30	28.80	30.20	27.60	32.20	28.20	33.30	29.50	27.90	34.00	36.20	27.80	32.70	28.20	31.40	*0.00			
[25] *C. lucius *	22.10	21.00	21.80	22.30	20.70	22.20	23.60	21.10	24.60	23.80	24.20	24.30	23.90	21.70	23.10	21.00	22.60	25.30	23.10	22.00	25.30	22.20	24.20	31.10	*N/A		
[26] *P. banksi *	16.60	15.80	15.70	17.90	14.80	14.30	17.70	17.60	25.60	19.60	22.80	17.20	20.60	14.50	24.20	14.10	14.60	26.70	23.50	14.90	24.00	18.20	23.80	30.60	23.40	*0.00	
[27] *P. smedleyi *	11.90	12.80	12.90	15.70	10.40	12.20	16.00	18.30	24.30	18.20	24.70	14.70	24.70	14.90	24.50	12.90	10.90	25.60	25.00	13.90	27.50	18.50	24.80	29.40	21.70	17.00	*0.00

**Table 4:** Comparison of divergence distance (K2P percent) calculated using K2P model.

Comparison of K2P distances within groups	Number of groups included in comparison	Minimum K2P percentage (%)	Maximum K2P percentage (%)	Mean K2P percentage (%)
Within species	24 species	0.00%	0.90%	0.16%
Within genera (among species)	4 genera*	1.70%	10.10%	6.03%
Within family (among genera)	2 families^†^	4.50%	15.70%	10.10%

*Channa, Hemibagrus, Puntioplites, Rasbora.

^†^Cyprinidae, Pangasiidae.

**Table 5:** List of standardised coefficients of canonical variables based on all *Channa striata *populations surveyed in Peninsular Malaysia. Measurements in “bold” and “italic” indicate the greatest contribution of characters to each root. The variables are corresponding to Truss Network Measurement illustrated in Figure 2.

Variable	Standardised coefficients for canonical variables					
	Root 1	Root 2	Root 3	Root 4	Root 5	Root 6
c2	***−1.41184***	0.499825	0.398591	0.054463	0.119042	−**0.238883**
f2	0.47170	0.408845	***−0.615390***	−0.168215	−0.230770	**−0.211542**
b2	0.28101	−0.278591	−0.284479	0.174256	−0.42253	**0.458817**
a10	0.41539	0.096533	−0.367550	−0.432404	0.259111	**−0.268992**
c3	0.66377	***−0.858751***	0.014077	0.472265	−0.250660	**−0.080690**
a5	0.36065	0.314906	−0.225384	−0.008893	0.267024	**0.198322**
e2	0.37393	0.331761	−0.266962	***0.681100***	−0.058005	**−0.360663**
b4	−0.19458	0.132053	0.252415	−0.497241	−0.523356	**0.359599**
SnL	−0.40295	−0.046967	0.003316	−0.148526	***0.532470***	**−0.133889**
b3	−0.47636	0.368229	***−0.728886***	0.429959	0.008980	**−0.328389**
a8	0.08207	−0.149248	0.373287	−0.265477	0.015430	**−0.064601**
b1	−0.43399	−0.523266	−0.084456	−0.507840	−0.286558	**−0.003799**
SnW	0.04462	0.228839	0.184555	0.652408	−0.226785	**−0.053505**
e1	***0.67757***	−0.152426	0.587134	***0.740656***	0.442494	***0.845807***
d1	−0.46682	0.329635	0.231088	−0.655794	−0.133296	**−0.085119**
g2	0.43378	−0.040481	0.309560	0.4676630	−0.394074	***0.556584***
g4	−0.40523	−0.077577	0.026801	−0.205765	0.396488	**0.091170**
h2	−0.48672	0.340375	−0.166280	0.050386	0.055264	**−0.023362**
a3	0.27658	0.063105	0.280636	0.288647	0.163754	**0.117652**
a6	−0.25723	0.396127	0.321013	0.032397	−0.299612	**0.211321**
d2	0.07330	***−0.585637***	0.007170	−0.075163	−0.137986	**−0.503075**
c1	0.17793	0.309891	−0.142353	−0.043706	***0.811599***	**−0.243137**
a4	0.25777	0.062941	−0.042803	−0.302267	0.331285	**−0.248307**
g3	−0.25389	−0.259770	−0.346391	0.242604	−0.036979	**0.115660**
a12	0.03025	0.253880	0.294079	−0.172015	−0.278936	**0.015935**
g1	−0.20226	0.291106	−0.012690	0.010235	−0.403767	**−0.146930**
f1	0.14296	−0.046102	0.320983	−0.248611	0.212844	**−0.372136**
h3	−0.14463	−0.158514	−0.212149	−0.032284	−0.166438	**0.353353**
a9	0.08448	0.017800	−0.010266	−0.270678	0.233016	**0.126766**
tl	0.07500	−0.141272	−0.182243	0.040925	0.012208	**0.065811**

Eigenvalue	14.89929	4.705745	3.882054	1.482290	0.334059	**0.148039**
Cum. Prop.	**0.58540 (58.54%)**	**0.770291 (18.49%)**	**0.922818 (15.25%)**	**0.981058 (5.82%)**	**0.994183 (1.31%)**	**1.000000 (0.58%)**

**Table 6:** List of standardised coefficients of canonical variables based on all *Channa striata *populations surveyed across entire Malaysia. Measurements in “bold” and “italic” indicate the greatest contribution of characters to each root. The variables are corresponding to Truss Network Measurement illustrated in Figure 2.

Variable	Standardised coefficients for canonical variables			
	Root 1	Root 2	Root 3	Root 4
e2	***2.1872***	***3.91106***	−0.20063	0.21126
c2	0.4818	−0.84864	***−1.70885***	0.08811
a5	−0.8042	−2.37991	1.35470	−0.82680
b1	***2.2417***	−0.37858	0.06038	−0.61243
a10	0.0702	0.13883	−0.14192	0.04250
f2	−1.0685	−0.79491	0.75069	−0.53315
a4	−0.0686	−2.99113	0.23824	***−1.09478***
g4	−2.0462	−0.56517	−0.82163	−1.07446
c3	1.0960	3.83485	−1.40422	0.20379
d1	−2.1132	***−5.06293***	1.15619	0.83281
SnL	1.7179	0.77327	−1.08747	0.41713
a3	−2.0655	0.90394	−0.30638	1.06254
b4	−1.6337	0.72442	0.09383	***1.44342***
g2	0.1400	1.89031	−0.14720	0.51732
a6	−2.0150	0.96549	0.42037	−0.75643
h2	1.4260	−2.70404	0.60591	0.13971
e1	1.2073	0.65500	0.18580	0.41229
g3	1.3471	1.36426	−0.57506	−0.92315
a9	−0.1035	−0.83578	0.18981	0.35164
a12	0.2179	−0.71198	0.24452	0.91279
a8	−0.5912	1.89025	***−1.70222***	0.92930
c1	0.9959	0.06944	1.27592	−0.77418
d2	1.3882	−0.48643	0.69451	−0.86227
SnW	−0.3964	−0.58803	0.77795	−0.50801
a1	−1.4941	−0.67406	0.28236	−0.67413
tl	0.9918	−0.12765	−0.75142	0.35329
a7	−0.7121	0.48801	0.72751	0.40213

Eigenvalue	222.9919	50.48606	19.21761	6.94981
Cum. Prop.	0.7442 (74.42%)	0.91267 (16.85%)	0.97681 (6.41%)	1.00000 (2.32%)
